# Novel functions of the anion exchanger AE4 (SLC4A9)

**DOI:** 10.1007/s00424-023-02899-5

**Published:** 2024-01-09

**Authors:** Helga Vitzthum, Catherine Meyer-Schwesinger, Heimo Ehmke

**Affiliations:** Institute of Cellular and Integrative Physiology, Center for Experimental Medicine, University Medical Center Hamburg-Eppendorf, Martinistrasse 52, 20246 Hamburg, Germany

**Keywords:** Acid, Base, Kidney, AE4, SLC4A9, Pendrin, SLC26A4, β-intercalated cell, Sodium reabsorption, Plasma volume, Alkalosis, Acidosis

## Abstract

The kidney plays a crucial role in acid-base homeostasis. In the distal nephron, α-intercalated cells contribute to urinary acid (H^+^) secretion and β-intercalated cells accomplish urinary base (HCO_3_^-^) secretion. β-intercalated cells regulate the acid base status through modulation of the apical Cl^-^/HCO_3_^-^ exchanger pendrin (SLC26A4) activity. In this review, we summarize and discuss our current knowledge of the physiological role of the renal transporter AE4 (SLC4A9). The AE4, as cation-dependent Cl^-^/HCO_3_^-^ exchanger, is exclusively expressed in the basolateral membrane of β-intercalated cells and is essential for the sensing of metabolic acid-base disturbances in mice, but not for renal sodium reabsorption and plasma volume control. Potential intracellular signaling pathways are discussed that might link basolateral acid-base sensing through the AE4 to apical pendrin activity.

## Introduction

**Fig. 1:**
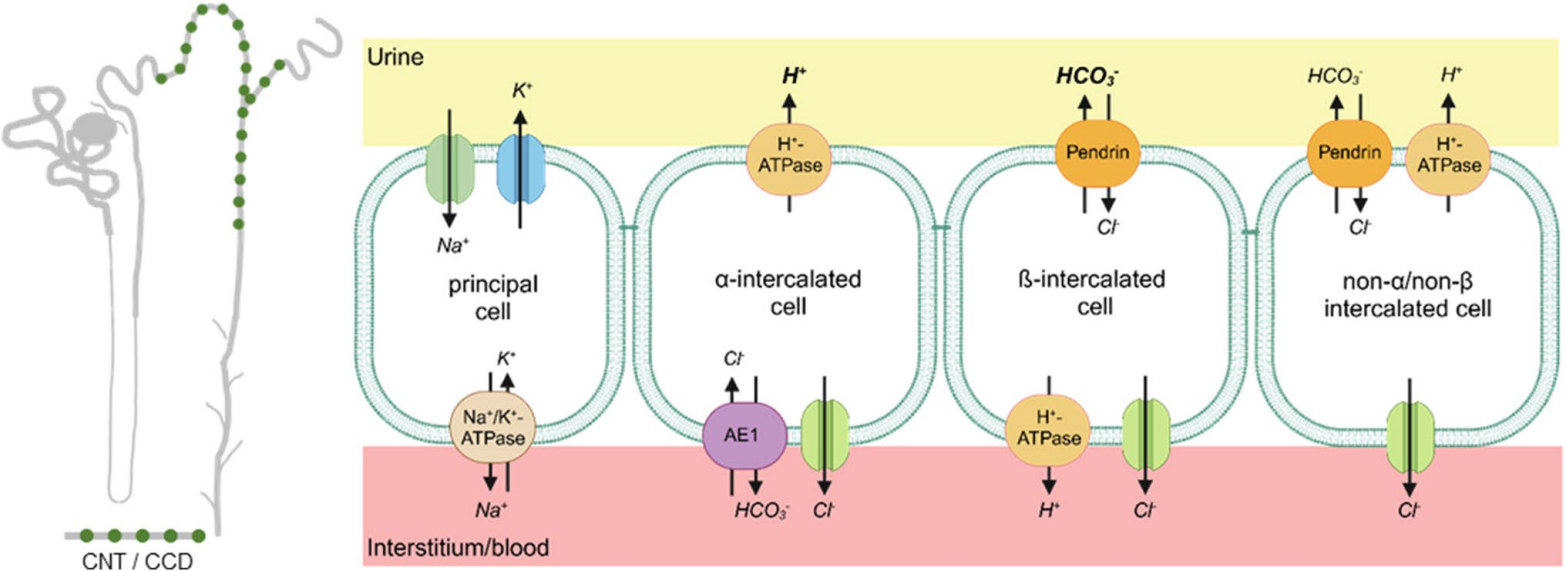
Functionally different cell types of the connecting tubule (CNT) and cortical collecting duct (CCD). The principal cells (PCs) are energized by the Na^+^/K^+^-ATPase and mediate Na^+^ reabsorption via the epithelial Na^+^ channel (ENaC) and K^+^ secretion via the K^+^ channel (ROMK). The intercalated cells (ICs) are energized by an H^+^-ATPase, which is apically expressed in α-ICs and localized to the basolateral membrane in β-ICs. The α-ICs secrete acid (H^+^) into the urine, whereas the β-ICs are base (HCO_3_^-^) secreting cells. The function of the non-α/non-β ICs, which express pendrin and H^+^-ATPase in the apical membrane, is less clear

In the kidney, the distal part of the nephron plays a crucial role for electrolyte, water, and acid-base homeostasis. To fulfill this task, in the connecting tubule (CNT) and the cortical collecting duct (CCD), functionally and morphologically distinct epithelial cell types, the so-called principal cells (PCs) and the intercalated cells (ICs) (Fig. 1), are present [79, 55, 57]. In contrast to PCs, the ICs are not energized by the Na^+^/K^+^-ATPase, but rather by a H^+^-ATPase [13]. Hence, the secondary transport in ICs depends on the activity of the basolateral or apical localized H^+^-ATPase. In the so-called α-ICs, the H^+^-ATPase is localized to the apical membrane, and together with the basolaterally localized Cl^-^/HCO_3_^-^ exchanger AE1 (SLC4A1), these cells contribute to acid (H^+^) secretion into the urine. Contrasting α-ICs, the β-ICs cells express the H^+^-ATPase in the basolateral plasma membrane. The β-ICs secrete base (HCO_3_^-^) through the apically located Cl^-^/HCO_3_^-^ exchanger pendrin (SLC26A4) into the urine. By this, the HCO_3_^-^ secretion of β-ICs is linked to H^+^ and Cl^-^ reabsorption. A third type of ICs (the non-α/non-β intercalated cells) has also been described, which expresses the H^+^-ATPase and pendrin at the apical membrane. The physiological function of the non-α/non-β ICs is not well defined, but it has been proposed that these cells may mediate net Cl^-^ reabsorption [30, 54, 76, 79].

Due to their properties and location within the nephron, the α- and β-ICs play a central and decisive role for acid-base homeostasis. Upon systemic acidosis, α-ICs are activated, and β-ICs are deactivated to maximize urinary acid excretion. In contrast, in the setting of metabolic alkalosis, the transport rate of β-ICs increases, whereas the transport rate of α-ICs decreases [74, 49, 45, 23], resulting in urinary base secretion. It was even reported that a long lasting acidosis may drive the conversion of β-ICs to α-ICs to maximize urinary acid excretion [1]. These adjustments in the ICs transport rate contribute significantly to acid-base homeostasis and help maintain blood pH within narrow limits.

Despite the central role of α- and β-ICs for systemic acid-base balance, the mechanisms and the signaling pathways that regulate the transport activity of the ICs are not yet fully understood. Several receptors have been found to influence the activity of ICs. For example, the proton-activated G protein-coupled receptor GPR4 impacts on α-ICs activity [63], the insulin receptor-related receptor (IRR) affects β-ICs activity [18], and the hormone secretin stimulates HCO_3_^-^ secretion of β-ICs via its receptor SCTR [5]. As metabolic acidosis and alkalosis are characterized by a change in plasma HCO_3_^-^ concentration [4], a direct influence of a derailed extracellular electrolyte composition on the activity of β-ICs is likely [12]. However, the question of how acid base status is detected systemically or locally by ICs is not clear. Here, we review the current knowledge regarding the transporter AE4 (SLC4A9) and its critical role in controlling β-ICs activity upon metabolic alkalosis and acidosis.

### Localization of AE4

Described for the first time in 2001, the transporter AE4 (SLC4A9) exhibits a very restricted and tissue specific expression pattern with high expression levels in the kidney of rat [32], rabbit [68], and human [37, 43]. Additionally, low levels of AE4 expression have been described in other tissues such as the salivary glands and the gastro-intestinal tract [32, 68].

**Fig. 2:**
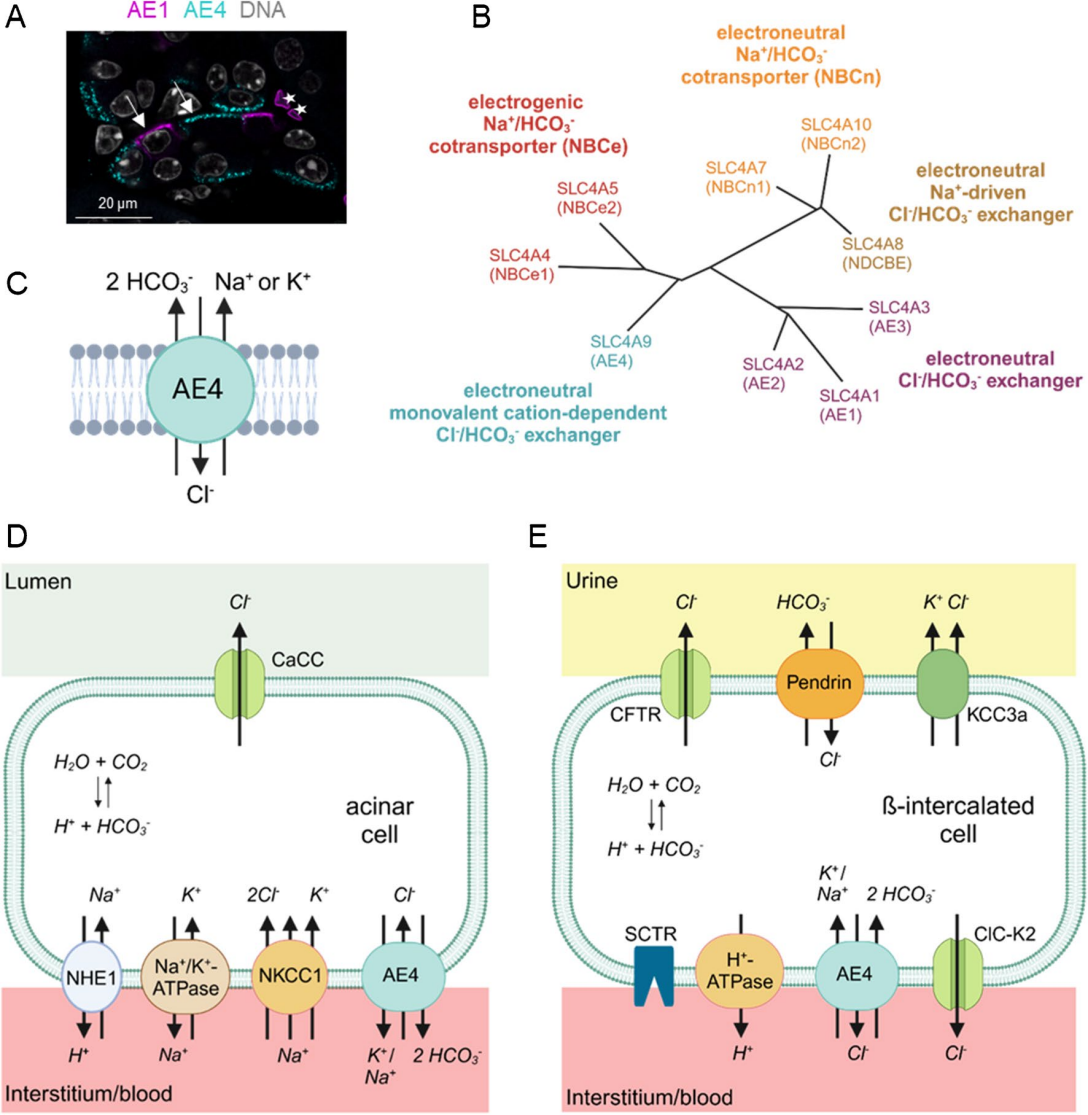
Localization, transport properties, and physiological function of AE4 (SLC4A9). **A** Localization of AE1 (SLC4A1) and AE4 (SLC4A9) in mouse kidney (arrows, basolateral expression; stars, red blood cells; methods as described in [73], the gpAE1 antibody was a generous gift from CA Wagner). **B** Phylogenetic tree of SLC4 family HCO_3_^-^ transporters (adapted from [38]). **C** AE4 mediates an electroneutral, cation-dependent Cl^-^/HCO_3_^-^ exchange (or Cl^-^/CO_3_^2-^). In addition to Na^+^, other monovalent cations such as K^+^ can be transported by AE4. The simple 1:1:2 transport stoichiometry is depicted [47]. **D** Simplified scheme of salivary gland cell adapted from [70]. Together with NKCC1 (SLC12A2), AE4 contributes to the high intracellular Cl^-^ concentration that enables apical Cl^-^ secretion via a Ca^2+^-dependent Cl^-^ channel (CaCC). **E** Basolateral (interstitium/blood) and apical (urine) transporters and receptors of β-ICs shown to be directly or indirectly involved in HCO_3_^-^ secretion (pendrin (SLC26A4), AE4 (SLC4A9) [73], *SCTR* secretin receptor [5]*, ClC-K2* chloride channel [61] [25], *KCC3a* K^+^/Cl^-^ cotransporter [20], *CFTR* cystic fibrosis transmembrane conductance regulator [9])

In the kidney, transcriptomic and proteomic data show that AE4, similar to pendrin, is exclusively present in the distal nephron [33, 36, 15]. RNA and protein expression analyses do not provide entirely consistent data concerning the precise localization of AE4 in the CNT/CCD. As such, single cell transcriptomics reported the highest AE4 expression in β-ICs, but also in murine [17] and human α-ICs [57], whereas principal cells showed no AE4 expression. Immunohistochemical approaches confirmed the presence of AE4 in the basolateral membrane of murine and human β-ICs [13, 49, 41, 73]. Additionally, some pendrin-negative cells have been described to express AE4, which might represent α-ICs [32, 26, 41]. However, own immunohistochemical data shown here could not discern any AE4 expression in AE1 expressing α-ICs (Fig. 2A), indicating that AE4 protein expression is confined to β-ICs. Whether AE4 is also expressed by the non-α/non-β ICs is still open to debate. Therefore, based on currently available evidence, the transporter AE4 appears to be functional only in the β-ICs, despite a broader RNA expression.

### Transport properties of AE4

AE4 belongs to the solute carrier family 4 (SLC4), which comprises 10 family members in mammals (SLC4A1-5 and SLC4A7-11). With the exception of the transporter SLC4A11, all members mediate transmembrane transport of base (HCO_3_^−^ or CO_3_^2-^), either through an exchange with Cl^-^ or via the cotransport with Na^+^ (Fig. 2B) [50, 52, 42, 38, 82]. The Cl^-^/HCO_3_^-^ exchangers include the Na^+^-independent AE1 (SLC4A1), AE2 (SLC4A2), AE3 (SLC4A3), and the Na^+^-driven Cl^-^/HCO_3_^-^ exchanger NDCBE (SLC4A8). Since the Na^+^/HCO_3_^-^ cotransporters (NBC) transport HCO_3_^-^ and Na^+^ in different stoichiometry, the transport of the NBCs can be either electrogenic (NBCe: SLC4A4 and SLC4A5) or electroneutral (NBCn: SLC4A7 and SLC4A10).

The functional properties of AE4 (SLC4A9) have long been controversial. On the one hand, it was reported that AE4 mediates Cl^-^/HCO_3_^-^ exchange [68, 32]. On the other hand, it was also suggested that AE4 represents a Na^+^/HCO_3_^-^ cotransporter [53, 13]. Based on studies in salivary gland acinar cells, it was recently shown that AE4 is an electroneutral, monovalent cation-dependent Cl^-^/HCO_3_^-^ exchanger [46, 47]. Since AE4 is not cation-selective, it can work as a Na^+^-dependent as well as a K^+^-dependent Cl^-^/HCO_3_^-^ exchanger (Fig. 2C) [47]. Additionally, under Cl^-^-free conditions, AE4 can exert Na^+^/HCO_3_^-^ cotransporter-like activity [47].

### Role of AE4 in salivary glands

In Cl^-^ secreting acinar cells of the salivary gland, the AE4 significantly contributes to the high intracellular Cl^-^ concentration ([Cl^-^]_i_), which is above the equilibrium potential of Cl^-^ (E_Cl_). As water and electrolyte secretion into the acinar lumen depends on Cl^-^ outflow across the apical membrane of acinar cells, the Cl^-^ uptake into the cells is a prerequisite for appropriate saliva production (Fig. 2D) [70]. The basolateral Cl^-^ uptake mainly depends on the Na^+^-K^+^-2Cl^-^ cotransporter 1 (NKCC1), but apparently also requires AE4 activity [46]. The intriguing observation was made that agonist stimulated salivary saliva secretion was markedly reduced in AE4 knockout (*AE4*^*-/-*^) mice [41]. This was explained by reduced basolateral Cl^-^ uptake in exchange for HCO_3_^-^.

It is worth to note that AE4 is bidirectional like other secondary transporters and its direction of transport strongly depends on the external physiological conditions [82]. Depending on extra- and intracellular concentrations of Cl^-^, HCO_3_^-^, Na^+^, or K^+^, the direction of transport by AE4 can change. Hence, the AE4 can either function as a Cl^-^ loader (transport of Cl^-^ from extra- to intracellular) or as a Cl^-^ extruder (transport of Cl^-^ from intra- to extracellular) [47]. Based on in vivo findings [46, 70], it was shown that in the Cl^-^ secreting salivary gland acinar cells, AE4 mediates Cl^-^ influx and HCO_3_^-^/K^+^(or Na^+^) efflux (Fig. 2D) [47, 70]. In these cells, the Cl^-^ and HCO_3_^-^ gradients across the basolateral membrane are crucial for the direction of transport of AE4, as the outward-directed K^+^ gradient and the inward-directed Na^+^ gradient are almost identical [47].

The direction of transport of AE4 in the HCO_3_^-^ secreting β-ICs is currently not resolved and still requires direct experimental evidence. In contrast to salivary gland acinar cells, β-ICs are primarily energized by an H^+^-ATPase [13]. Stimulation of the H^+^-ATPase impacts on the [HCO_3_^-^] gradient and thereby alters the driving force for the transport of AE4. In addition, the [Cl^-^] gradient in the β-ICs depends on the activities of several transporters which are affected by acid-base imbalances, such as CFTR [6], KCC3a [21], and pendrin. However, the assumption that AE4 can function as a Cl^-^ extruder and a HCO_3_^-^ loader in these cells is very appealing (Fig. 2E) [19]. Furthermore, a change in extracellular electrolyte concentrations during metabolic acid-base imbalances could influence the [HCO_3_^-^] gradient across the basolateral membrane. Accordingly, one may speculate that an increase or decrease in extracellular [HCO_3_^-^] upon alkalosis and acidosis, respectively, influences the rate and/or direction of transport of AE4 in β-ICs.

### AE4 appears not to be essential for sodium reabsorption and plasma volume regulation

Numerous studies have demonstrated the importance of the β-ICs for transcellular Cl^-^ reabsorption in the CNT/CCD and hence for the regulation of plasma volume [78, 72, 28, 29, 66, 77]. Accordingly, ablation or mutations in the *slc26a4* gene, which impair or eliminate pendrin activity, also affect blood pressure regulation in both rodents [66] and humans [29]. In addition, Cl^-^ reabsorption mediated by the Cl^-^/HCO_3_^-^ exchanger pendrin (SLC26A4) is not only activated upon alkalosis but also by dietary Cl^-^ depletion [71, 78, 31, 28]. The upregulation of pendrin under salt restricted conditions is mediated, at least in part, by an activation of the mineralocorticoid receptor (MR) in β-ICs [58, 59, 27, 2].

In addition, it was reported that β-ICs carry out transcellular Na^+^ reabsorption. Based on in vivo studies in mice harboring a genetic ablation of the Na^+^-dependent anion exchanger NDCBE (*Slc4a8*^*-/-*^), it was suggested that Na^+^ uptake across the apical membrane (from the urine into the β-ICs) is mediated through the parallel action of pendrin and NDCBE [34, 60]. As the expression of NDCBE in the renal cortex and particularly in β-ICs is still controversial [17, 81, 35, 16], alternative apical Na^+^ uptake pathways such as the Na^+^/H^+^ exchanger NHE2 (SLC9A2) have also been suggested [81]. Similarly, the exit route for the absorbed Na^+^ on the basolateral side of the β-ICs is also not understood. In principal cells, the Na^+^/K^+^-ATPase provides the basolateral exit pathway for Na^+^, which is taken up by the apically localized epithelial Na^+^ channel ENaC (Fig. 1). Since abundance [17] and activity [56] of the Na^+^/K^+^-ATPase is much lower in β-ICs than in principal cells, it was assumed that a basolaterally located Na^+^/HCO_3_^-^ cotransporter might mediate the Na^+^ extrusion in these cells [13]. The observation that Na^+^-dependent HCO_3_^-^ absorption is markedly reduced in CCDs isolated from kidneys of AE4 knockout mice (*AE4*^*-/-*^) implied that AE4 may represent the major basolateral exit pathway for Na^+^ in β-ICs [13], a concept that is now widely accepted and included in many reviews [51, 79, 11, 77]. Extensive in vivo analyses, however, rebut an essential role of AE4 for renal Na^+^ handling and hence vascular volume control [73]. In comparison to pendrin (*Slc26a4*^*-/-*^) [78] or NDCBE (*Slc4a8*^*-/-*^) [60] knockout mice, AE4 knockout (*AE4*^-/-^) mice neither showed a salt-losing phenotype nor intravascular volume constriction in the unchallenged or in the salt-restricted setting [73]. In both *AE4*^-/-^ and wildtype mice, exposure to dietary salt restriction resulted in a prompt and comparable decrease of urinary Na^+^ and Cl^-^ excretion. Consequently, plasma volume and the activation of the renin-angiotensin II-aldosterone-system (RAAS) did not differ between genotypes. Furthermore, as no compensatory activation of other renal Na^+^ reabsorption pathways such as the Na^+^/Cl^-^ cotransporter NCC or the epithelial Na^+^ channel ENaC were present in *AE4*^*-/-*^ mice upon salt restriction, contrasting observations made in the NDCBE knockout (*slc4a8*^*-/-*^) mice, it is unlikely that the AE4 is part of an essential pathway for renal Na^+^ reabsorption. Future studies are needed to elucidate if and how transcellular Na^+^ transport can be made possible in β-ICs and if so, which basolateral Na^+^ efflux pathway is present in β-ICs.

### AE4 is essential for acid-base homeostasis

The β-ICs play a critical role for overcoming alkalosis of various origin as they are unique in their ability to actively secrete HCO_3_^-^ into the urine [11]. Metabolic alkalosis, characterized by elevated blood pH, decreased plasma [Cl^-^], and increased plasma [HCO_3_^-^] [4], can be caused by excessive base supply or loss of acids. In addition, electrolyte imbalances, such as hypochloremia or hypokalemia, or a diminished effective circulating volume, can induce a metabolic alkalosis [40, 24, 75]. The importance of the β-ICs in protection against alkalosis is well documented in models with genetic modification of β-IC transporters, channels or receptors (Fig. 2E). Upon alkali loading pendrin knockout (*Slc26a4*^*-/-*^) mice develop severe metabolic alkalosis [71, 72]. Similarly, patients with Pendred syndrome, a disease caused by a mutation in the pendrin gene (*Slc26a4/Pds*), show hypochloremic metabolic alkalosis when exposed to an increased alkali load or a thiazide treatment [80]. Recent studies have shown that loss of the Cl^-^ channel CFTR [69, 8, 7] or the secretin receptor (SCTR) [5] also impairs renal HCO_3_^-^ secretion and causes metabolic alkalosis upon perturbation.

**Fig. 3:**
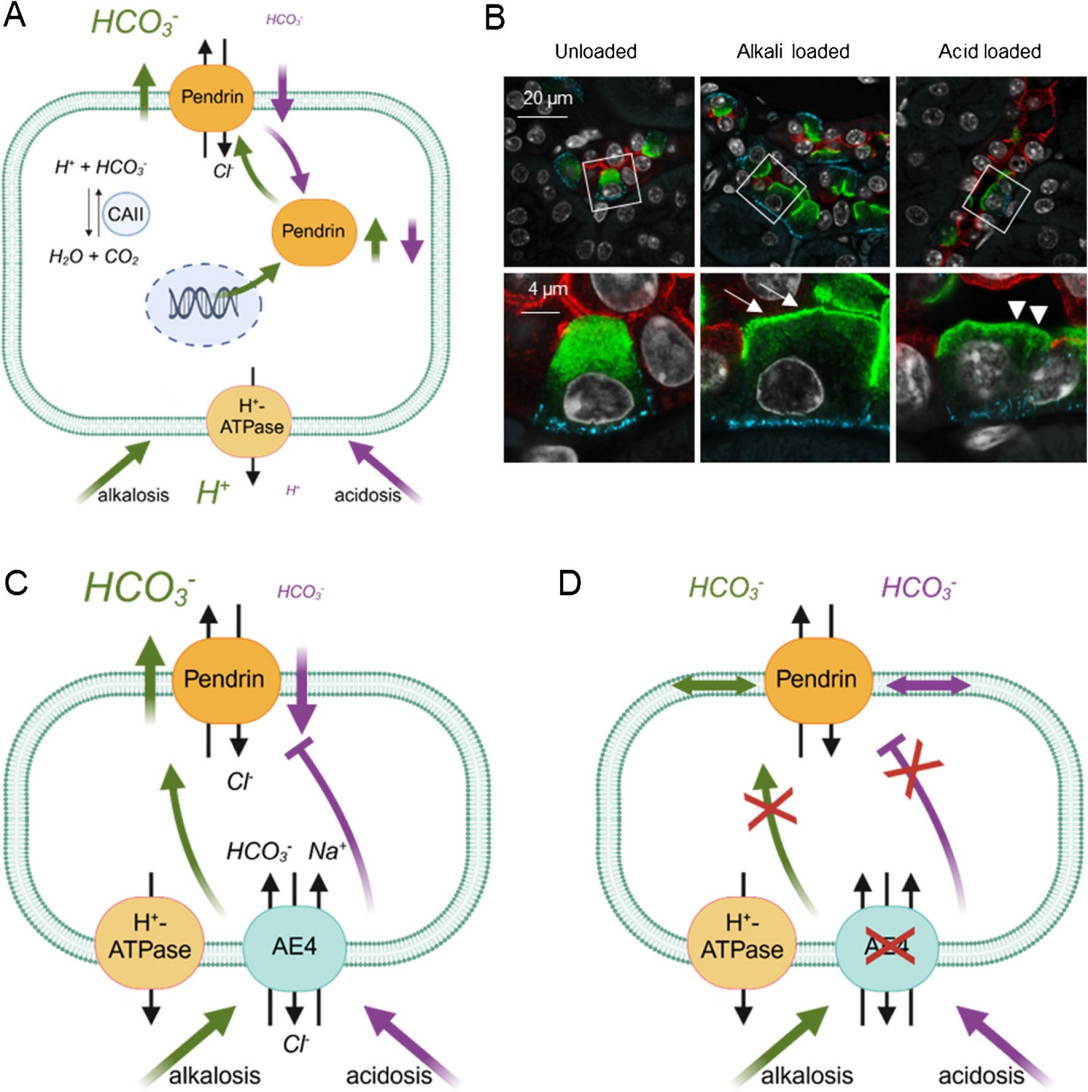
AE4 dependent regulation of pendrin upon metabolic alkalosis and acidosis. **A** Alkalosis causes upregulation of pendrin mRNA and protein levels, induces translocation into the apical membrane, and increases pendrin activity. Acidosis decreases pendrin activity and expression levels and induces withdrawal of pendrin from the apical membrane. HCO_3_^-^ is provided by hydration of CO_2_ by carbonic anhydrases (CAII). **B** Confocal micrographs depicting translocation of pendrin (green) to the apical membrane (arrows) and withdrawal from the membrane, resulting in a reduction of pendrin cap size (arrow heads). AE4 (blue) and the principal cell marker Aquaporin 2 (red), methods as described in [73]). **C** AE4 couples pendrin activity to the acid-base status. Thereby, the urinary HCO_3_^-^ secretion is increased upon alkalosis and decreased upon acidosis. **D** AE4 ablation uncouples pendrin activity from the acid base status. Thus, urinary HCO_3_^-^ secretion is not adapted to acid base imbalances

Upon alkalosis an enhanced HCO_3_^-^ secretion into the urine is achieved by β-ICs through several cellular adaptive responses (Fig. 3A), comprising a higher pendrin transport rate [9], the recruitment of pendrin to the apical membrane by translocation from the subapical region (Fig. 3B) [72, 45], and an enhanced pendrin mRNA and protein synthesis [74, 23]. Opposite responses take place upon metabolic acidosis. As such, pendrin is withdrawn from the apical membrane and a downregulation of pendrin protein takes place, leading to reduced urinary base (HCO_3_^-^) secretion upon acidosis (Fig. 3A,B) [49]. These regulatory steps are crucial for adjusting urinary HCO_3_^-^ secretion to systemic acid-base imbalances. Accordingly, both too low or too high levels of pendrin activity can cause, sustain, or aggravate metabolic alkalosis or acidosis, respectively [11].

The importance of AE4 in regulating β-IC activity and thus acid-base balance is highlighted by recent findings in AE4 knockout mice (*AE4*^*-/-*^) loaded with acid or with base [73]. Continuously base loaded wildtype mice showed enhanced activation of pendrin and urinary HCO_3_^-^ secretion (Fig. 3C), which caused a rapid normalization of the acid base status. In contrast, continuously base loaded AE4 knockout mice developed metabolic alkalosis, which severely aggravated over time. This life-threatening, hypochloremic metabolic alkalosis was the result of a failure to activate pendrin and as a consequence, impaired HCO_3_^-^ secretion into the urine (Fig. 3D). Adding to the severe phenotype in AE4 knockout mice, renal pendrin abundance and the number of pendrin-positive cells were even reduced upon prolonged base loading. In summary, current findings strongly support that AE4 is an essential element of the acid-base sensor in β-ICs, controlling pendrin activity during metabolic acid base imbalances.

In β-ICs, the apically secreted HCO_3_^-^ emerges from the conversion of CO_2_ to HCO_3_^-^ catalyzed by carbonic anhydrase, coupled with the basolateral extrusion of H^+^ via H^+^-ATPase (Fig. 3A). As has already been noted for other HCO_3_^-^ secreting epithelia such as the pancreatic duct [62], it is conceivable that a high HCO_3_^-^ secretion rate in β-ICs also requires an additional source of HCO_3_^-^, namely uptake by a basolateral transporter. Due to its transport properties, AE4 may serve as an HCO_3_^-^ entry route to facilitate increased pendrin transport rates. The new conception that AE4 is also a sensor is supported by several findings [73]. First, the knockout of AE4 not only prevents enhanced HCO_3_^-^ secretion upon base loading, but also abolishes the translocation of pendrin to the apical membrane and abrogates the necessitated increase of pendrin abundance. Second, acid loaded AE4 knockout mice do not show the expected β-IC response to acidosis that comprises the removal of pendrin from the apical membrane and the downregulation of pendrin abundance. The resulting unadjusted high pendrin activity results in a sustained urinary HCO_3_^-^ secretion (Fig. 3D) and in an aggravation of metabolic acidosis in AE4 knockout mice, similar to other animal models with pendrin overactivity [39, 11]. Collectively, these findings highlight the importance of a concurrent activation of α-ICs and inactivation of β-ICs for a rapid correction of metabolic acidosis.

Interestingly, the inability of AE4 knockout mice to alter pendrin activity secondary to acid-base loading is independent of the salt content of the diet, as AE4 knockout leads to a decoupling of pendrin activity and acid base status in Cl^-^ restricted (i.e., NaCl-deficient diet with NaHCO_3_ supplement) or Cl^-^ replete settings (i.e., normal NaCl diet with NaHCO_3_ supplement; see Suppl. Fig. 5 in reference [73]). However, the severity of the acid base disorder strongly depends on the NaCl content of the diet. The severe and life-threatening aggravation of metabolic alkalosis in base loaded AE4 knockout mice was only observed under Cl^-^ restricted conditions. When AE4 knockout mice were challenged with a base load under Cl^-^ replete conditions, the acid base status normalized after a few days, even though pendrin activation was absent. The observation that Cl^-^ repletion is sufficient to correct persistent metabolic alkalosis of various origins was made a long time ago, but the underlying renal mechanism remained unsolved [22, 40]. Low Cl^-^ delivery to the distal nephron could reduce the driving force for HCO_3_^-^/Cl^-^ exchange by pendrin and therefore functionally prevent sufficient urinary HCO_3_^-^ excretion by pendrin. In addition, Cl^-^ deficiency may also impact on HCO_3_^-^ and H^+^ handling in other nephron segments. Further studies are required to elucidate the changes in renal HCO_3_^-^ transport induced by higher Cl^-^ availability.

### Candidate intracellular signaling molecules

The intracellular pathways that link basolateral AE4 solute transport to apical pendrin activity in β-ICs are unknown. The altered blood electrolyte concentrations characteristic for metabolic alkalosis and acidosis are thought to change the HCO_3_^-^ influx and the Cl^-^ efflux through AE4 in β-ICs. Whether an increased uptake or decreased export of both anions is associated with a significant change of their intracellular concentration remains to be evaluated. However, both anions have been shown to affect regulatory cytosolic enzymes such as the HCO_3_^-^-regulated soluble adenylyl cyclase (sAC), which is an important source of cAMP [14], and the Cl^-^-sensitive (with-no-lysine) kinase WNK4 [64].

An alteration of intracellular HCO_3_^-^ concentration influences the activity of the soluble adenylyl cyclase (sAC) and hence the intracellular level of cAMP [67]. The sAC, which is present in the intercalated cells [44], has already been proposed as a candidate pH sensor in the kidney and other tissues such as the epididymis [12]. According to recent findings, cAMP is an important intracellular signaling molecule in β-ICs [10]. The hormone secretin enhances the activity of pendrin and urinary HCO_3_^-^ secretion [9, 5] through binding to its G protein-coupled receptor in the basolateral membrane of β-ICs (SCTR, Fig. 2E) [6]. Previous studies showed that secretin stimulation causes cAMP formation in renal tubule suspensions [48] and that cAMP induces translocation of pendrin to the membrane in opossum kidney proximal (OKP) cells, which express pendrin after transfection [3]. Increased abundance of pendrin was also observed after stimulation of microdissected CNTs and CCDs with forskolin or 8-br-cAMP [65]. Hence, it is possible that cAMP may be as critical for AE4-mediated pendrin stimulation as it is for mediating the cellular responses to secretin. It is important to note, however, that cAMP has not yet been shown to cause translocation of pendrin into the apical membrane of β-IC or to induce a change in pendrin gene or protein expression in vivo.

As a second possible link between the basolateral AE4 transport and apical pendrin activity, changes in cytosolic Cl^-^ concentrations, together with the with-no-lysine kinase WNK4, could be an element of the intracellular signaling network in β-ICs. The WNK4 is part of the WNK family, which is characterized by its unique Cl^-^ sensing properties [64]. An AE4-mediated reduction in intracellular Cl^-^ concentration in alkalosis could lead to WNK4 activation. The important role of WNK4 for the β-ICs is already highlighted by a mouse model of pseudo-hypoaldosteronism type II (PHAII) [39]. Mice carrying the PHAII mutated WNK4 transgene (*TgWnk4*^*PHAII*^ mice), which results in higher WNK4 activity, not only exhibit hypertension and hyperkalemia, but also acidosis. Genetic ablation of pendrin in the *TgWnk4*^*PHAII*^ mice corrected acid base status [39], demonstrating that the renal tubular acidosis is caused by pendrin hyperactivity. Whether metabolic acid-base disturbances modulate intracellular Cl^-^ concentrations of β-ICs in an AE4-dependent manner and thus alter WNK4 activity remains to be determined.

## Concluding remarks

Recent findings have brought significant progress in elucidating the transport properties and physiological functions of AE4 (SLC4A9). Two essential roles of this electroneutral, cation-dependent Cl^-^/HCO_3_^-^ exchanger are now evident. In the salivary gland, the exchanger contributes so saliva secretion by loading the acinar cells with Cl^-^. In the kidney, AE4 protein expression is restricted to the basolateral membrane of β-ICs. While AE4 does not play a major role in sodium reabsorption for plasma volume control, the competence of β-ICs to respond appropriately to metabolic acid-base disturbances depends entirely on AE4. Future efforts should aim at unraveling the intracellular signaling molecules that convey the AE4-dependent control of pendrin and thus β-cell activity.

## Data Availability

Not applicable
